# Clinical, health systems and neighbourhood determinants of tuberculosis case fatality in urban Blantyre, Malawi: a multilevel epidemiological analysis of enhanced surveillance data

**DOI:** 10.1017/S0950268821001862

**Published:** 2021-09-02

**Authors:** McEwen Khundi, Peter MacPherson, Helena R. A. Feasey, Rebeca Nzawa Soko, Marriott Nliwasa, Elizabeth L. Corbett, James R. Carpenter

**Affiliations:** 1 Malawi-Liverpool-Wellcome Trust Clinical Research Programme, Blantyre, Malawi; 2 London School of Hygiene and Tropical Medicine, London, UK; 3 Department of Clinical Sciences, Liverpool School of Tropical Medicine, Liverpool, UK; 4 Helse Nord TB Initiative, College of Medicine, University of Malawi, Blantyre, Malawi

**Keywords:** Epidemiology, HIV, multilevel modelling, statistics, treatment outcomes, tuberculosis

## Abstract

We investigated whether household to clinic distance was a risk factor for death on tuberculosis (TB) treatment in Malawi. Using enhanced TB surveillance data, we recorded all TB treatment initiations and outcomes between 2015 and 2018. Household locations were geolocated, and distances were measured by a straight line or shortest road network. We constructed Bayesian multi-level logistic regression models to investigate associations between distance and case fatality. A total of 479/4397 (10.9%) TB patients died. Greater distance was associated with higher (odds ratio (OR) 1.07 per kilometre (km) increase, 95% credible interval (CI) 0.99–1.16) odds of death in TB patients registered at the referral hospital, but not among TB patients registered at primary clinics (OR 0.98 per km increase, 95% CI 0.92–1.03). Age (OR 1.02 per year increase, 95% CI 1.01–1.02) and HIV-positive status (OR 2.21, 95% CI 1.73–2.85) were also associated with higher odds of death. Model estimates were similar for both distance measures. Distance was a risk factor for death among patients at the main referral hospital, likely due to delayed diagnosis and suboptimal healthcare access. To reduce mortality, targeted community TB screening interventions for TB disease and HIV, and expansion of novel sensitive diagnostic tests are required.

## Introduction

In 2019, tuberculosis (TB) was the leading cause of death from a single infectious disease in the world, causing more deaths than HIV/AIDS and malaria combined [1]. Slow progress has been made in reducing TB mortality, and the World Health Organization's (WHO) End TB strategy target of achieving a 90% reduction in TB deaths between 2015 and 2030 [1] is unlikely to be met.

In the WHO Africa (AFRO) region, TB treatment success rates (cured or completed treatment) in 2017 were 86% for HIV-negative people and 78% for HIV-positive people [1]. Despite these improvements from 2007 (when overall TB treatment success was estimated to be 79% in Africa), a considerable fraction of people with TB symptoms are delayed in accessing TB diagnosis and care at health facilities [1, 2]. The reasons for delayed access to treatment are complex and multi-layered, but include health care-seeking behaviours, clinical and geographical factors, and suboptimal care quality in health facilities [3].

Distance to health facilities is a well-recognised access barrier to prompt diagnosis [4–7]. Individuals who live far from health facilities are at an increased risk of unfavourable health outcomes [4–6] and at an increased risk of death on TB treatment [8–10]. However, there is limited evidence about associations between distance and death on TB treatment in urban African settings.

Using prospectively collected data from people initiating TB treatment at health facilities in urban Blantyre, Malawi in the era of high antiretroviral therapy (ART) coverage for HIV, we hypothesised that people initiating TB care at facilities at greater distances from their homes might be at greater risk of death on TB treatment compared to people who lived nearer to clinics. Additionally, as accurate measurement of clinic distance may be challenging under routine programmatic conditions, we compared distance measurement using two approaches that could be used by health planners and epidemiologists: Cartesian distance and shortest road network distance.

## Methods

### Study setting and design

Blantyre District is located in the southern region of Malawi. Blantyre city, in the centre of the district, has large areas of densely-populated informal settlements. Blantyre's 2018 census population was 1 264 304 [11] and HIV prevalence was 18% [12].

### Blantyre enhanced TB monitoring and evaluation

In Malawi, patients diagnosed with TB register to receive treatment at primary health care centres and hospitals. In Blantyre, TB registration clinics include one referral hospital (Queen Elizabeth Central Hospital (QECH)), one large private church-supported clinic (Mlambe), three private clinics and seven government public primary health care clinics [13]. QECH is a tertiary referral hospital for the whole southern region of Malawi and offers inpatient care including TB diagnosis and treatment [13].

In a joint project between the Malawi-Liverpool-Wellcome Trust Clinical Research Programme, Blantyre District Health Office and the Malawi National TB Control Programme (NTP), TB Officers (a cadre of health worker employed by the Ministry of Health of Malawi) received training to strengthen the TB surveillance system in Blantyre. Since 2015, TB Officers stationed at TB treatment clinics in Blantyre provided TB treatment, HIV testing and linkage to treatment in accordance with Malawi guidelines. They further recorded TB registration data into NTP TB registers and additionally recorded individual-level clinical, socio-demographic and household data using an electronic data collection application (ePaL), which we have previously developed and validated (18). Using ePaL, TB officers obtained global positioning satellite (GPS) coordinates for the location of TB patients’ households (18). A spot sputum sample was also collected from all patients starting TB treatment (if patients were able to produce sputum) that underwent smear microscopy and mycobacteria growth indicator tube culture. On a quarterly basis, study and TB programme registers were reconciled.

TB outcomes were defined according to mutually-exclusive WHO TB treatment outcome definition guidelines [14], with patients classified as either cured, completed treatment, treatment failed, confirmed died, lost to follow-up or not evaluated (typically because still on treatment). The outcome of interest for this analysis was confirmed death on TB treatment; we did not follow-up patients to confirm death. The analysis was limited to study participants that were registered for treatment from 1 January 2015 to 30 December 2018.

### Statistical analysis

#### Baseline characteristics

We compared the characteristics of participants, recorded at TB treatment registration, between those who died during TB treatment and those who were alive at the end of treatment. For categorical variables, we calculated percentages and used the *χ*^2^ test for comparisons; for continuous variables, we calculated medians (and interquartile ranges (IQR)), mean (and standard deviation) and compared between groups using the Kruskal–Wallis test.

#### Distance estimation

**Fig. 1.**
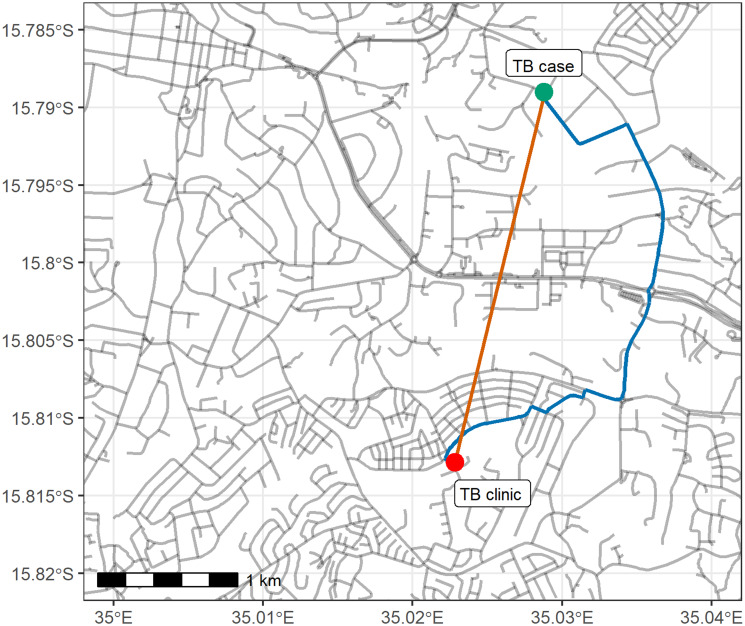
Illustration of the two methods for the measurement of household to clinic distance: Cartesian distance (2.7 km), shortest road network distance (4.5 km). *Note*: The patient place of residence is a randomly generated point for illustration and does not correspond to any patient in the dataset.

We estimated the distance from study participants’ households to their TB treatment initiation clinic using two approaches (Fig. 1). The first approach was to estimate the distance based on a ‘straight line’ distance (Cartesian distance). In the second approach, we used Blantyre urban road network downloaded from OpenStreetMap (OpenStreetMap Foundation) to calculate the shortest road network distance using the stplanr R package [15].

#### Statistical models

**Fig. 2.**
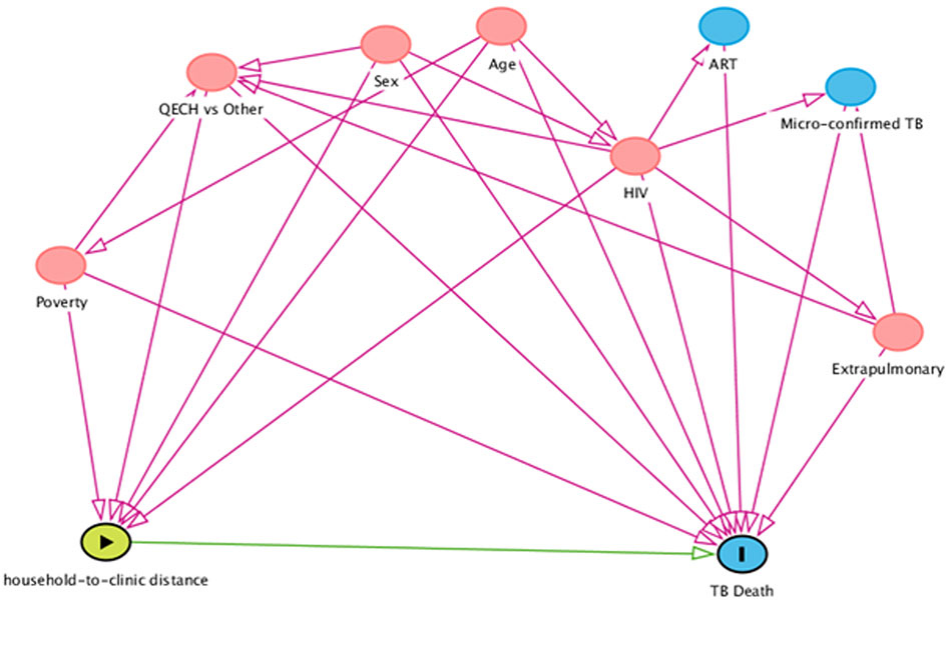
Directed acyclic graph (causal diagram). Illustrating the relationship between distance to TB clinic and risk of death on TB treatment and other covariates. The variables sex, age, HIV, Queens Elizabeth hospital registration (QECH) *vs*. registration at other clinics and poverty were selected as the minimum adjustment set.

To estimate the causal relationship between clinic distance and TB case fatality, we constructed a directed acyclic graph (Fig. 2). The minimum adjustment set of confounders identified from the DAG were: sex, age, HIV, hospital admission (QECH) and household wealth score. We included a term for the interaction between household to clinic distance and the clinic at which TB registration occurred (QECH, the city's referral hospital, *vs*. other primary health care centres). This was because QECH is a large referral hospital known to have a patient population with more advanced TB disease, more complicated TB disease and patients that are likely to have travelled a longer distance compared to patients that attend other clinics.

Using asset ownership data, we created a household wealth score variable, calculated using a proxy means test developed for urban populations from the Malawi Integrated Household Survey [16]. Individuals for whom components needed to calculate their wealth score were missing; their wealth score was imputed using multiple interval imputation, with the observed wealth score providing the lower bound.

We constructed Bayesian multi-level logistic regression models (Equation 1) to investigate the association between participants’ household to clinic distance and their risk of death, adjusting for confounders and an interaction between household to clinic distance and clinic of registration. Weakly regularising priors, refined by inspecting prior predictive plots, were assigned to model intercepts and slopes. Model convergence was assessed by visual inspection of trace plots, effective sample numbers and Gelman–Rubin statistics.1
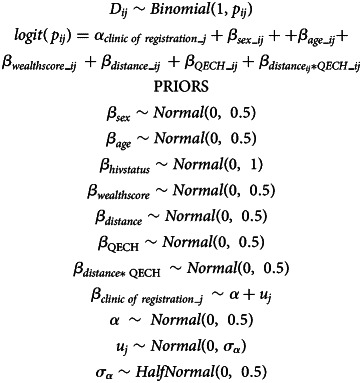
where *D_ij_* indicates death on TB treatment for the *i*^th^ patient in clinic *j*, sex indicates sex (male or female), age indicates age in years, hivstatus indicates HIV status (positive or negative), wealthscore indicates household wealth score, distance indicates distance to clinic in kilometres, and QECH indicates whether participant registered for treatment at QECH or the other primary health centres, distance×QECH is the interaction effect between distance and QECH and clinic of registration indicates registering for treatment at the *j*^th^ TB treatment registration health centre.

Four thousand samples with a warm up of 600 iterations were drawn from each model posterior distribution using Markov Chain Monte Carlo methods implemented within Stan via the brms R package [17]. Posterior means and 95% credible intervals (CI) were calculated on the log-odds scale and were exponentiated to give odds ratios. In sensitivity analysis, we refitted models recoding participants who were lost to follow-up or transferred out during TB treatment as having died since it was possible that some of these patients had actually died [18]. Additionally, we restricted the analysis to those with microbiologically-confirmed TB. To investigate the predictive accuracy of the two distance estimates, we used models that just had distance and the interaction effect of distance and registering for TB treatment at QECH. We plotted the difference in the predicted probability of death for the 100 patients with the greatest difference in distance from the two measurement methods. Analysis was conducted using R version 3.5.2 (R Foundation for Statistical Computing, Vienna).

#### Ethical considerations

Ethical approval was granted by the London School of Hygiene and Tropical Medicine (16228) and the College of Medicine, University of Malawi Research Ethics Committee (P.12/18/2556). Participants provided oral consent to participate in TB surveillance with a waiver for written consent granted by both research ethics committees.

## Results

### Baseline characteristics

A total of 4461/5199 (85.8%) patients that initiated TB treatment at the 12 study clinics were included in this analysis. In total, 64/4461 (1.4%) had a missing TB outcome and were excluded from the primary analysis (Appendix Table A1). Among participants that had treatment outcomes, 479/4397 (10.9%) died while taking TB treatment, and 258/4397 (5.9%) were reported as lost to follow-up or transferred out (Table 1). The percentage of participants who died did not substantially differ across the study years (*P* = 0.313).

**Table 1.** Characteristics for notified tuberculosis cases in urban Blantyre Malawi, 2015–2018

	Alive (*N* = 3918)	Died (*N* = 479)	Total (*N* = 4397)	*P*-value
Year of registration				0.313
2015	860 (89.1%)	105 (10.9%)	965 (100.0%)	
2016	1187 (89.5%)	139 (10.5%)	1326 (100.0%)	
2017	1238 (88.0%)	169 (12.0%)	1407 (100.0%)	
2018	633 (90.6%)	66 (9.4%)	699 (100.0%)	
Gender				0.934
Female	1432 (89.1%)	176 (10.9%)	1608 (100.0%)	
Male	2486 (89.1%)	303 (10.9%)	2789 (100.0%)	
Age, years				<0.001
Median (IQR)	35.0 (28.0, 41.0)	37.0 (30.0, 45.0)	35.0 (28.0, 42.0)	
Mean (SD)	35.1 (13.4)	38.3 (13.7)	35.4 (13.4)	
HIV status				<0.001
Negative	1337 (94.4%)	79 (5.6%)	1416 (100.0%)	
Positive	2581 (86.6%)	400 (13.4%)	2981 (100.0%)	
TB classification				<0.001
Extrapulmonary TB	1375 (85.5%)	233 (14.5%)	1608 (100.0%)	
Pulmonary TB	2543 (91.2%)	246 (8.8%)	2789 (100.0%)	
Micro confirmed TB				<0.001
Not microbiologically-confirmed TB	2064 (86.6%)	318 (13.4%)	2382 (100.0%)	
Microbiologically-confirmed TB	1854 (92.0%)	161 (8.0%)	2015 (100.0%)	
Distance km (Cartesian)				<0.001
Median (IQR)	3.8 (2.0, 5.7)	4.4 (2.9,6.8)	3.8 (2.1, 5.9)	
Mean (SD)	4.3 (3.1)	4.9 (3.1)	4.3 (3.1)	
Distance km (road network)				<0.001
Median (IQR)	5.1 (3.1, 7.5)	5.8 (4.1, 9.0)	5.2 (3.2, 7.7)	
Mean (SD)	5.7 (3.7)	6.5 (3.7)	5.8 (3.7)	
Wealth score^a^				0.461
Missing	3446	417	3863	
Median (IQR)	2.5 (2.2, 3.0)	2.6 (2.2, 3.1)	2.5 (2.2, 3.0)	
Mean (SD)	2.6 (0.5)	2.6 (0.6)	2.6 (0.5)	
Clinic of registration				<0.001
Other clinics	2063 (92.6%)	164 (7.4%)	2227 (100.0%)	
Queen Elizabeth Central Hospital	1855 (85.5%)	315 (14.5%)	2170 (100.0%)	

HIV, human immunodeficiency virus; km, kilometres; (Q1, Q3) interquartile range; SD, standard deviation; TB, tuberculosis.

a Wealth score, household wealth developed using household asset ownership.

More deaths were reported among participants who initiated TB treatment at QECH (315/2170, 14.5%) than in the other 11 TB clinics (164/2227, 7.4%, *P* < 0.001; Table 1). Similar percentages of men (303/2789, 10.9%) and women (176/1608, 10.9%, *P* = 0.934) died during TB treatment. The median age of participants who died was older (median: 37 years, IQR: 30.0–45.0) compared to those who were alive at the end of TB treatment (35 years, IQR: 28.0–41.0, *P* < 0.001). A higher proportion of deaths occurred in patients who were HIV-positive (400/2981, 13.4%) compared to HIV-negative (79/1416, 5.6%, *P* < 0.001). Participants who did not have a microbiologically-confirmed TB (318/2382, 13.4%) were considerably more likely to die compared to participants with either sputum smear, Xpert or culture-positive disease (161/2015, 8.0%, *P* < 0.001).

The median road network household to clinic distance (median: 5.2 km, IQR: 3.2–7.7) was consistently higher than the Cartesian household to clinic distance estimates (median: 3.8 km, IQR: 2.1–5.9). Both household to clinic distance estimates showed that patients who died on TB treatment lived further from clinics where they initiated TB treatment than those that were alive at the end of TB treatment. For the road network distance measure, those who died on TB treatment had greater clinic distances (median: 5.8 km, IQR: 4.1–9.0) compared to participants who were still alive at TB treatment completion (median: 5.1 km, IQR: 3.1–7.5, *P* < 0.001). Likewise, those who died on TB treatment had a longer Cartesian distance (median: 4.4 km, IQR: 2.9–6.8) *vs*. participants who were still alive at the end of TB treatment (median: 3.8 km, IQR: 2.0–5.7, *P* < 0.001). Patients who did not live within geographically-mapped areas of the city were excluded 738/5199 (14.2%) because we did not have data to ascertain their household location, and so could not calculate their household to clinic distance (Appendix Table A1).

### Unadjusted analysis of the association of household to clinic distance and risk of death

TB patients who lived further from the clinic at which they registered for treatment had higher odds of death compared to those who lived nearer, whether measured by the road network distance method (odds ratio (OR) 1.05 per km increase, 95% CI 1.03–1.08) or the Cartesian distance method (OR 1.06 per km increase, 95% CI 1.03–1.09; Table 2).

**Table 2.** Statistical model results for the main analysis, notified TB cases in urban Blantyre, Malawi from 2015–2018

Variable	Unadjusted OR (95% CI)	Adjusted OR (95% CI)	Adjusted OR network distance^a^ (95% CI)	Adjusted OR Cartesian distance^b^ (95% CI)
Sex (male)	0.99 (0.81–1.21)		1.06 (0.87–1.29)	1.06 (0.87–1.30)
Age (years)^c^	1.02 (1.01–1.02)		1.02 (1.01–1.02)	1.02 (1.01–1.02)
HIV positive	2.53 (2.00–3.25)		2.21 (1.73–2.85)	2.21 (1.73–2.86)
Wealth score^c^^,^^d^	1.40 (0.98–2.04)		1.15 (0.80–1.73)	1.14 (0.80–1.72)
Queen Elizabeth Central Hospital	2.10 (1.74–2.56)			
Network distance (km)^c^	1.05 (1.03–1.08)			
Cartesian distance (km)^c^	1.06 (1.03–1.09)			
Road network distance		1.02 (0.99–1.06)	0.98 (0.92–1.03)	
Queen Elizabeth Central Hospital		1.93 (1.57–2.38)	2.00 (0.93–4.25)	
Network distance (km)×Queen Elizabeth Central Hospital		1.02 (0.96–1.08)^e^	1.07 (0.99–1.16)	
Cartesian distance		1.03 (0.99–1.07)		0.97 (0.89–1.04)
Queen Elizabeth Central Hospital		1.91 (1.55, 2.35)		2.00 (0.92–4.27)
Cartesian distance (km)×Queen Elizabeth Central Hospital		1.03 (0.96–1.11)^f^		1.09 (1.00–1.21)
*Clinic of registration intercept*			0.06 (0.04–0.11)	0.06 (0.04–0.11)
*Clinic of registration intercept standard deviation*			0.67 (0.32–1.18)	0.69 (0.33–1.19)

km, Kilometres; OR, odds ratio.

The analysis was done for all the data at once (*N* = 4397) using Bayesian multi-level logistic regression models.

a,b Adjusted for sex (male *vs*. female), age in years, HIV status (HIV-positive *vs*. HIV-negative), wealth score the household wealth score, TB treatment registration at Queen Elizabeth Central Hospital (QECH) *vs*. the other health care facilities, distance household to clinic distance in kilometres, distance (km)×QECH interaction effect of QECH and household to clinic distance and a term for the random intercept of clinic of registration.

c Age in years, household to clinic distance and household wealth score were centred by subtracting the mean.

d Wealth score of household wealth developed using household asset ownership.

e,f Adjusted for TB treatment registration at QECH *vs*. other health care facilities, household to clinic distance, and the interaction effect of QECH and household to clinic distance.

### Unadjusted analysis for confounders

In unadjusted analysis, each one year increase in age was associated with a 2% increase (OR 1.02, 95% CI 1.01–1.02) in the odds of death. Being HIV-positive was associated with a 2.5-times (OR *vs*. HIV-negative status 2.53, 95% CI 2.0–3.25) increase in the odds of death on TB treatment. TB patients who registered at QECH had two times higher (OR 2.10, 95% CI 1.74–2.56) odds of death compared to people registering at the other primary clinics. Each one-unit increase in wealth score was associated with a 40% (OR 1.40, 95% CI 0.98–2.04) increase in the odds of death.

### Adjusted analysis for household to clinic distance models

Greater household to clinic distance (road network) had a higher odds of death in TB patients registered at QECH (OR 1.07 per km increase, 95% CI 0.99–1.16), but was not associated with odds of death in TB patients registered at other clinics (OR 0.98 per km increase, 95% CI 0.92–1.03). The average risk of death varied between clinics by 6% (95% CI 4–11%) holding categorical variables at their base level and continuous variables at their average value, with a standard deviation of 0.67 (0.32–1.18; Table 2).

For the model containing Cartesian distance to clinic, model coefficients and uncertainty bounds were very similar to those from the road distance model, with distance from household to clinic of treatment registration associated with higher odds of death in TB patients registered at QECH (OR 1.09 per km increase, 95% CI 1.0–1.21), but was not associated with odds of death on TB treatment in patients registered at other clinics (OR 0.97 per km increase, 95% CI 0.89–1.04).

### Comparison of model predicted risk of death among 100 participants with biggest difference between their road network distance and Cartesian distance estimates

**Fig. 3.**
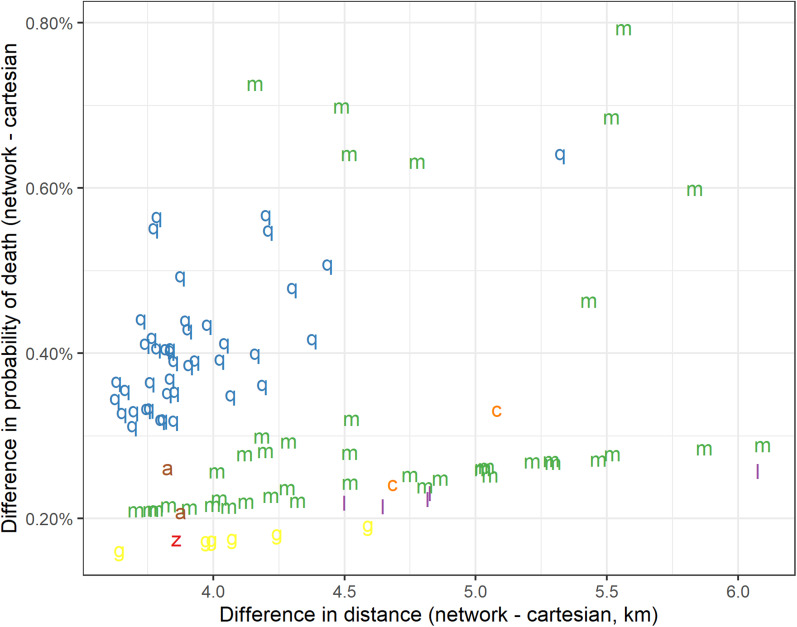
Plot of difference between shortest road network and Cartesian distance, *vs*. fitted probability of death, for the 100 notified TB cases with the largest distance differences. q, Queen Elizabeth Central Hospital; m, Mlambe Private Hospital; l, Limbe Health Centre; g, Bangwe Health Centre; z, Zingwangwa Health Centre; a, Blantyre Adventist Hospital; c, Chilomoni Health Centre.

The majority of the 100 TB patients with the greatest difference in distance between the two household to clinic distance measurements registered for treatment at QECH or Mlambe private clinic (Fig. 3). For these participants who had the greatest measurement difference, there was, as expected, a trend of increased difference in probability of death with a greater difference between the two distance measures. The greatest differences were seen at Mlambe private clinic, reflecting greater distances travelled by road. However, the maximum difference in the predicted probability of death over these 100 patients was only 0.79%.

### Sensitivity analysis

When we reclassified TB patients who were lost to follow-up or had transferred out during TB treatment as having died, model estimates were very similar to our primary analysis (Table 3). Analysis restricted to participants with microbiologically-confirmed disease did not alter our model conclusion (Table 4).

**Table 3.** Statistical model results for the sensitivity analysis: patients with loss to follow-up or transfer out treatment status recoded as having died, in urban Blantyre, Malawi, 2015–2016

Variable	Unadjusted OR (95% CI)	Adjusted OR (95% CI)	Adjusted OR network distance^a^ (95% CI)	Adjusted OR Cartesian distance^b^ (95% CI)
Sex (male)	1.10 (0.94–1.28)		1.06 (0.87–1.29)	1.06 (0.87–1.29)
Age (years)^c^	1.01 (1.01–1.02)		1.02 (1.01–1.03)	1.02 (1.01–1.03)
HIV positive	1.76 (1.47–2.11)		2.21 (1.73–2.85)	2.21 (1.73–1.84)
Wealth score^c^^,^^d^	1.39 (0.97–2.04)		1.15 (0.80–1.72)	1.15 (0.80–1.72)
Queen Elizabeth Central Hospital	1.44 (1.23–1.68)			
Network distance (km)^c^	1.03 (1.01–1.05)			
Cartesian distance (km)^c^	1.01 (0.98–1.05)			
Road network distance		1.02 (0.99–1.04)	0.98 (0.92–1.03)	
Queen Elizabeth Central Hospital		1.37 (1.16–1.64)	2.02 (0.94–4.31)	
Network distance (km)×Queen Elizabeth Central Hospital		1.01 (0.96–1.07)^e^	1.07 (0.99–1.16)	
Cartesian distance		1.02 (0.99–1.05)		0.97 (0.89–1.04)
Queen Elizabeth Central Hospital		1.37 (1.17–1.61)		2.00 (0.92–4.26)
Cartesian distance (km)×Queen Elizabeth Central Hospital		1.02 (0.96–1.09)^f^		1.09 (1.00–1.21)
*Clinic of registration intercept*			0.06 (0.04–0.11)	0.06 (0.04–0.11)
*Clinic of registration intercept standard deviation*			0.68 (0.33–1.17)	0.69 (0.33–1.19)

km, Kilometres; OR, odds ratio.

The analysis was done for all the data at once (*N* = 4397) using Bayesian multi-level logistic regression models with participants that defaulted and those that transferred out (*N* = 258) recoded as died.

a,b Adjusted for sex (male *vs*. female), age in years, HIV status (HIV-positive *vs*. HIV-negative), wealth score the household wealth score, TB treatment registration at Queen Elizabeth Central Hospital (QECH) *vs*. other health care facilities, distance household to clinic distance in kilometres, distance (km)×QECH interaction effect of QECH and household to clinic distance and a term for the random intercept of clinic of registration.

c Age in years, household to clinic distance and household wealth score were centred by subtracting the mean.

d Wealth score of household wealth developed using household asset ownership.

e,f Adjusted for TB treatment registration at QECH *vs*. other health care facilities, household to clinic distance, and the interaction effect of QECH and household to clinic distance.

**Table 4.** Statistical model results for the sensitivity analysis: restricted to patients with microbiologically-confirmed tuberculosis disease only in urban Blantyre Malawi, 2015–2018

Variable	Unadjusted OR (95% CI)	Adjusted OR (95% CI)	Adjusted OR network distance^a^ (95% CI)	Adjusted OR Cartesian distance^b^ (95% CI)
Sex (male)	0.93 (0.69–1.28)		0.99 (0.72–1.38)	0.99 (0.72–1.38)
Age (years)^c^	1.02 (1.00–1.03)		1.01 (1.00–1.03)	1.01 (1.00–1.03)
HIV positive	3.06 (2.12–4.59)		2.65 (1.79–4.06)	2.66 (1.78–4.03)
Wealth score^c^^,^^d^	1.85 (0.95–3.49)		1.63 (0.84–3.04)	1.62 (0.84–3.37)
Queen Elizabeth Central Hospital	2.22 (1.62–3.04)			
Network distance (km)^c^	1.07 (1.02–1.11)			
Cartesian distance (km)^c^	1.06 (0.98–1.14)			
Road network distance		0.97 (0.88–1.04)	0.97 (0.88–1.04)	
Queen Elizabeth Central Hospital		1.67 (0.86–3.42)	1.67 (0.86–3.42)	
Network distance (km)×Queen Elizabeth Central Hospital		1.14 (1.01–1.29)^e^	1.14 (1.01–1.29)	
Cartesian distance		1.01 (0.94–1.08)		0.98 (0.87–1.06)
Queen Elizabeth Central Hospital		1.73 (1.18–2.43)		1.66 (0.86–3.37)
Cartesian distance (km)×Queen Elizabeth Central Hospital		1.14 (1.00–1.29)^f^		1.16 (1.01–1.35)
*Clinic of registration intercept*			0.04 (0.02–0.09)	0.04 (0.02–0.0.09)
*Clinic of registration intercept standard deviation*			0.45 (0.02–1.22)	0.43 (0.02–1.20)

km, Kilometres; OR, odds ratio.

The analysis was done using *N* = 2015 participants that had a microbiologically confirmed TB diagnosis using Bayesian multi-level logistic regression models.

a,b Adjusted for sex (male *vs*. female), age in years, HIV status (HIV-positive *vs*. HIV-negative), wealth score the household wealth score, TB treatment registration at Queen Elizabeth Central Hospital (QECH) *vs*. the other health care facilities, household to clinic distance in kilometres, distance (km)×QECH interaction effect of QECH and household to clinic distance and a term for the random intercept of clinic of registration.

c Age in years, household to clinic distance and household wealth score were centred by subtracting the mean.

d Wealth score of household wealth developed using household asset ownership.

e,f Adjusted for TB treatment registration at QECH *vs*. other health care facilities, household to clinic distance, and the interaction effect of QECH and household to clinic distance

## Discussion

Our primary hypothesis was that greater household to clinic distance would be associated with an increased risk of death for people starting treatment for TB in urban Blantyre, Malawi. The results of the pre-specified unadjusted analysis showed clinic distance was a risk factor for death after starting TB treatment. However, in adjusted analysis based on our causal graph, greater clinic distance was a risk factor for death only for participants initiating care at the tertiary referral hospital (QECH). Treatment initiation at the city's tertiary hospital, older age and HIV-positive status were important predictors of death whilst taking TB treatment. We additionally found moderate variation in the risk of death between treatment clinics that was not explained by TB patient clinical and socio-demographic characteristics, indicating that clinic quality of care might be an important residual determinant of treatment outcomes.

We used two different measures (Cartesian distance, and shortest road network distance) of household to clinic distance that could be applied by public health planners and epidemiologists, recognising that road network distance may be considerably more challenging to estimate under routine programmatic conditions. Our analysis found that models using either distance measurement reached very similar conclusions, and therefore we recommend that in low-resource settings, the simpler measurement (Cartesian distance) should likely be more appropriate for routine programme use.

For TB patients that were treated at the city's tertiary hospital (QECH), greater household to clinic distance might be a proxy for lack of access to quality health care service [8, 19]. Individuals who live in the peri-urban areas far from the centre of Blantyre have limited resources for transport, and may struggle to access timely tertiary-level health care compared to those living nearer to the centre of the city [20]. Delayed presentation for care will then result in admission to hospital with more advanced disease and at a higher risk of death [5, 21].

To reduce the high risk of death on TB treatment, prompt diagnosis and treatment for people presenting to health facilities is required [21, 22]. At health facilities in high HIV-prevalence settings, TB symptoms are common (up to 60% reporting at least one of the WHO four cardinal TB symptoms) [23]. Therefore, novel screening approaches – including triage testing using high sensitivity initial tests such as chest X-ray or C-reactive protein, followed by highly specific tests such as Xpert for those who triage test positive, require evaluation [5, 24].

QECH is a tertiary referral hospital, and most patients treated for TB at QECH will have previously sought diagnosis – likely on multiple occasions before referral – at a primary health centre [25]. Consequently, earlier diagnosis in primary health care could reduce hospital admission and potentially reduce mortality [1]. We need to improve the TB diagnostic capacity of primary health facilities in the outskirts of the city and ensure that primary health care facilities can make prompt referrals for hospital care where required [1, 5, 8].

In addition, there is a need to evaluate community interventions of TB screening in areas further away from the centre of the city (QECH) since prevalent cases identified in community interventions are usually diagnosed at an earlier stage of disease [1, 5]. Our previous work has shown that TB case notifications decline with greater distance from the centre of Blantyre city; this might be because of lack of access to quality health care, including TB diagnosis, at areas in the periphery of the city [20]. Health promotion activities to promote early treatment seeking, and quality improvement activities with health workers to support early diagnosis and reduce variation in practice between clinics could contribute to reduced TB case fatality, whilst strengthening universal healthcare provision within health facilities [1, 5].

Malawi has made tremendous progress towards achieving the WHO 90-90-90 HIV targets [26]: 90% of individuals with HIV are aware of their HIV status, 79% of individuals with a diagnosis are on ART and 72% of individuals on ART treatment have viral suppression [27]. Nevertheless, in this study, HIV-positive status remained strongly associated with an increased risk of death during TB treatment. Nearly all TB patients in this study were aware of their HIV-positive status, and 89% were taking ART at TB treatment initiation. Persistently high case fatality for HIV-positive people despite high ART coverage suggests that HIV-positive people have severe acute illness [28], have profound delays in TB diagnosis and treatment initiation [29, 30], or have virological failure to HIV treatment, which itself confers a high risk of death. In a study among HIV-positive people taking ART who were admitted to hospital in Zomba District, Malawi (32%) had virological treatment failure, and resistance to first-line ART drugs was near-universal [31]. Improvements in viral load monitoring and rapid treatment changes linked to supportive adherence interventions could reduce the number of patients that present with severe disease at the start of TB treatment and save more lives [31, 32]. Additionally, implementation of point-of-care HIV viral load monitoring for people living with HIV who are admitted to hospital could identify treatment failure earlier. Following WHO recommendations, the Malawi National HIV Programme has recommended that all people living with HIV take a Dolutegravir containing regimen, including by switching treatment regimens for those already taking [31, 33]. It will be important to investigate the effect of this change on TB case fatality among patients treated at primary clinics and in hospital (QECH) [33].

A key strength of this study was that we used prospectively collected data at TB registration through our citywide enhanced TB surveillance system. Our routine monitoring and evaluation show consistently very high agreement with national TB treatment registers. We additionally captured TB patients’ household GPS coordinates at the point of registration for TB treatment; most previous studies have attempted to retrospectively geolocate patients’ households using physical addresses, a method that is prone to error [34, 35]. Our analysis of household to clinic distance was analysed at a continuous scale rather than on a transformed categorical scale with arbitrary value cut-off points [36, 37]. To investigate the causal relationship between household to clinic distance and risk of death, we selected confounding variables for adjustment by constructing a directed acyclic graph [38]. Sensitivity analysis restricted to TB patients with microbiologically-confirmed TB and where patients who were lost to follow-up or transferred out to another clinic did not alter our findings. However, timing of death was not collected meaning survival analysis could not be done. A small number of TB patients may not have been able to accurately geolocate their household, or may have deliberately misidentified their household; monthly quality assurance checking of a 5% random sample of households attempted to mitigate this. While Appendix Table A1 indicates that TB patients registering for treatment who were residents in the areas of the city not mapped by our GPS had some different characteristics compared to those living in the mapped areas. Nevertheless, the main objective of this analysis was to predict TB case fatality using data obtained at treatment registration and there is no reason to believe that the underlying relationships should differ between those included and excluded.

In conclusion, in prespecified multilevel modelling using citywide enhanced surveillance data linked to our satellite GPS location system, we found that household to clinic distance was associated with increased risk of death for a patient starting TB treatment at the city's central hospital. This could be explained by barriers in accessing prompt diagnosis and treatment, and by variable quality of care at primary health facilities. HIV-positive status and older age remain important risk factors for death. Therefore, interventions that improve access to TB diagnosis through community-based active case finding, and improved quality of health facility TB screening, and prioritise HIV-positive people with potentially high levels of viral failure for TB screening and ART optimisation are required to reduce the unacceptably high case fatality.

## Data Availability

Data will be made available upon request.

## Appendix

**Table A1.** Characteristics for notified Tuberculosis cases in Urban Blantyre Malawi, 2015–2018. Comparing participants who were included in the study versus those who were not part of the study but were from Blantyre.

	In study (*N* = 4461)	Not in study (*N* = 738)	Total (*N* = 5199)
Year of registration			
2015	966 (21.7%)	149 (20.2%)	1115 (21.4%)
2016	1330 (29.8%)	191 (25.9%)	1521 (29.3%)
2017	1437 (32.2%)	222 (30.1%)	1659 (31.9%)
2018	728 (16.3%)	176 (23.8%)	904 (17.4%)
Gender			
Male	2827 (63.4%)	405 (54.9%)	3232 (62.2%)
Female	1634 (36.6%)	333 (45.1%)	1967 (37.8%)
Age, years			
Median (Q1, Q3)	35.0 (28.0, 42.0)	35.0 (21.3, 45.0)	35.0 (27.0, 42.0)
Mean (SD)	35.5 (13.4)	34.2 (19.0)	35.3 (14.4)
HIV status			
HIV negative	1428 (32.0%)	333 (45.1%)	1761 (33.9%)
HIV positive	3033 (68.0%)	405 (54.9%)	3438 (66.1%)
TB classification			
Extrapulmonary TB	1637 (36.7%)	466 (63.1%)	2103 (40.5%)
Pulmonary TB	2824 (63.3%)	272 (36.9%)	3096 (59.5%)
Micro confirmed TB			
Not microb-confirmed TB	2420 (54.2%)	621 (84.1%)	3041 (58.5%)
Microb-confirmed TB	2041 (45.8%)	117 (15.9%)	2158 (41.5%)
TB treatment outcome			
N-Miss	64	15	79
Cured	916 (20.8%)	31 (4.3%)	947 (18.5%)
Treatment completed	2715 (61.7%)	419 (58.0%)	3134 (61.2%)
Died	479 (10.9%)	94 (13.0%)	573 (11.2%)
Treatment failure	29 (0.7%)	2 (0.3%)	31 (0.6%)
Lost to follow up	258 (5.9%)	177 (24.5%)	435 (8.5%)
Clinic of registration			
Other clinics	2257 (50.6%)	113 (15.3%)	2370 (45.6%)
Queen Elizabeth Central Hospital	2204 (49.4%)	625 (84.7%)	2829 (54.4%)

Abbreviations: HIV, Human immunodeficiency virus; (Q1, Q3) interquartile range; SD standard deviation; TB tuberculosis

## References

[1] World Health Organization (2019) *Global Tuberculosis Report 2018*. WHO. Geneva.

[2] World Health Organization (2010) *Global Tuberculosis Control Report 2009*. Geneva.

[3] Heunis JC, (2017) Risk factors for mortality in TB patients: a 10-year electronic record review in a South African province. BMC Public Health 17, 38. 28061839 10.1186/s12889-016-3972-2 PMC5217308

[4] Mayer CM, (2019) Distance to clinic is a barrier to PrEP uptake and visit attendance in a community in rural Uganda. Journal of the International AIDS Society 22, e25276. 31037845 10.1002/jia2.25276 PMC6488759

[5] Wold Health Organisation (2013) Systematic Screening for Active Tuberculosis. Geneva.

[6] Momenyan S, (2018) Spatial inequalities and predictors of HIV/AIDS mortality risk in Hamadan, Iran: a retrospective cohort study. Epidemiology and Health 40, e2018038. 30081619 10.4178/epih.e2018038 PMC6232660

[7] O'Donnell O (2007) Access to health care in developing countries: breaking down demand side barriers. Cadernos de Saude Publica 23, 2820–2834. 18157324 10.1590/s0102-311x2007001200003

[8] Tripathy JP, (2013) Is physical access an impediment to tuberculosis diagnosis and treatment? A study from a rural district in North India. Public Health Action 3, 235–239. 26393036 10.5588/pha.13.0044 PMC4463123

[9] Fluegge K, (2018) Impact of geographic distance on appraisal delay for active TB treatment seeking in Uganda: a network analysis of the Kawempe Community Health Cohort Study. BMC Public Health 18, 798. 29940918 10.1186/s12889-018-5648-6 PMC6019214

[10] Cai J, (2015) Factors associated with patient and provider delays for tuberculosis diagnosis and treatment in Asia: a systematic review and meta-analysis. PLoS ONE 10, e0120088. Published online: 25 March 2015. doi: 10.1371/journal.pone.0120088. 25807385 PMC4373856

[11] National Statistical Office of Malawi (NSO) (2018) *Malawi Population Census Preliminary Report*. Zomba.

[12] Malawi Ministry of Health. Malawi Population-Based HIV Impact Assessment (MPHIA) 2015–2016: Final Report. Lilongwe, Ministry of Health. October 2018.

[13] Makwero MT (2018) Delivery of primary health care in Malawi. African Journal of Primary Health Care and Family Medicine 10, 1799. 10.4102/phcfm.v10i1.1799 PMC6018651 29943590

[14] World Health Organization (WHO) (2014) TB case and treatment outcome definitions. World Health Organization; Published online: 2014.

[15] Lovelace R and Ellison R (2018) stplanr: A Package for Transport Planning. The R Journal 10, 7–23.

[16] Payongayong E, (2006) Simple household poverty assessment models for Malawi: proxy means test from the 1997–98 Malawi Integrated Household Survey. Lilongwe, Malawi: National Statistical Office, Government of Malawi.

[17] Bürkner P-C (2017) brms: an R package for Bayesian multilevel models using stan. Journal of Statistical Software 80, 1–28.

[18] Squire SB, (2005) ‘Lost’ smear-positive pulmonary tuberculosis cases: where are they and why did we lose them? International Journal of Tuberculosis and Lung Disease 9, 25–31. 15675546

[19] Secretary of Jan Swasthya Sahyog, Laux TS and Patil S (2018) Predictors of tuberculosis treatment outcomes among a retrospective cohort in rural, Central India. Journal of Clinical Tuberculosis and Other Mycobacterial Diseases 12, 41–47. 31720398 10.1016/j.jctube.2018.06.005 PMC6830133

[20] MacPherson P, (2019) Disparities in access to diagnosis and care in Blantyre, Malawi, identified through enhanced tuberculosis surveillance and spatial analysis. BMC Medicine 17, 21. 30691470 10.1186/s12916-019-1260-6 PMC6350280

[21] Virenfeldt J, (2014) Treatment delay affects clinical severity of tuberculosis: a longitudinal cohort study. BMJ Open 4, 4818. 10.1136/bmjopen-2014-004818 PMC4067883 24916087

[22] World Health Organization (2013) *Systematic screening for active tuberculosis. Principles and recommendations*. Geneva. 25996015

[23] Chihota VN, (2015) Missed opportunities for TB investigation in primary care clinics in South Africa: experience from the XTEND Trial. PLoS ONE 10, e0138149. 26383102 10.1371/journal.pone.0138149 PMC4575203

[24] van't Hoog AH, (2012) Screening strategies for tuberculosis prevalence surveys: the value of chest radiography and symptoms. PLoS ONE 7, e38691. 22792158 10.1371/journal.pone.0038691 PMC3391193

[25] Chikovore J, (2015) ‘For a mere cough, men must just chew Conjex, gain strength, and continue working’: the provider construction and tuberculosis care-seeking implications in Blantyre, Malawi. Global Health Action 8, e26292. Published online: 2015. doi: 10.3402/gha.v8.26292. PMC4382597 25833138

[26] United Nations Joint Programme on HIV/AIDS (UNAIDS) (2020) Joint United Nations Programme on HIV/AIDS. UNAIDS data 2020. Geneva, Switzerland; 436.

[27] United Nations Joint Programme on HIV/AIDS (UNAIDS). Malawi country HIV/AIDS factsheet 2019. Available at https://www.unaids.org/en/regionscountries/countries/malawi (Accessed 29 October 2020).

[28] Kwan C and Ernst JD (2011) HIV and tuberculosis: a deadly human syndemic. Clinical Microbiology Reviews 24, 351–376. 21482729 10.1128/CMR.00042-10 PMC3122491

[29] Nogueira BMF, (2018) Factors associated with tuberculosis treatment delay in patients co-infected with HIV in a high prevalence area in Brazil. PLoS ONE 13, e0195409. Published online: 1 April 2018. doi: 10.1371/journal.pone.0195409. 29624603 PMC5889181

[30] Gesesew H, (2016) The prevalence and associated factors for delayed presentation for HIV care among tuberculosis/HIV co-infected patients in Southwest Ethiopia: a retrospective observational cohort. Infectious Diseases of Poverty 5, 96. 27802839 10.1186/s40249-016-0193-y PMC5090949

[31] Gupta-Wright A, (2020) Virological failure, HIV-1 drug resistance, and early mortality in adults admitted to hospital in Malawi: an observational cohort study. The Lancet HIV8 7, e620–e628. 10.1016/S2352-3018(20)30172-7 PMC7487765 32890497

[32] Craik A, (2016) Challenges with targeted viral load testing for medical inpatients at Queen Elizabeth Central Hospital in Blantyre, Malawi. Malawi Medical Journal 28, 179–181. 28321282 10.4314/mmj.v28i4.6 PMC5348611

[33] World Health Organisation (2019) *Update of recommendations on first-and second-line antiretroviral regimens*. Geneva.

[34] Cegielski JP, (2013) Eliminating tuberculosis one neighborhood at a time. American Journal of Public Health 103, 1292–1300. 23078465 10.2105/AJPH.2012.300781 PMC3682594

[35] Robsky KO, (2020) Is distance associated with tuberculosis treatment outcomes? A retrospective cohort study in Kampala, Uganda. BMC Infectious Diseases 20, 406. 32527306 10.1186/s12879-020-05099-z PMC7291553

[36] Royston P, Altman DG and Sauerbrei W (2006) Dichotomizing continuous predictors in multiple regression: a bad idea. Statistics in Medicine 25, 127–141. 16217841 10.1002/sim.2331

[37] Altman DG and Royston P (2006) The cost of dichotomising continuous variables. British Medical Journal 332, 1080. 16675816 10.1136/bmj.332.7549.1080 PMC1458573

[38] Textor J, (2016) Robust causal inference using directed acyclic graphs: the R package ‘dagitty’. International Journal of Epidemiology 45, 1887–1894. 28089956 10.1093/ije/dyw341

